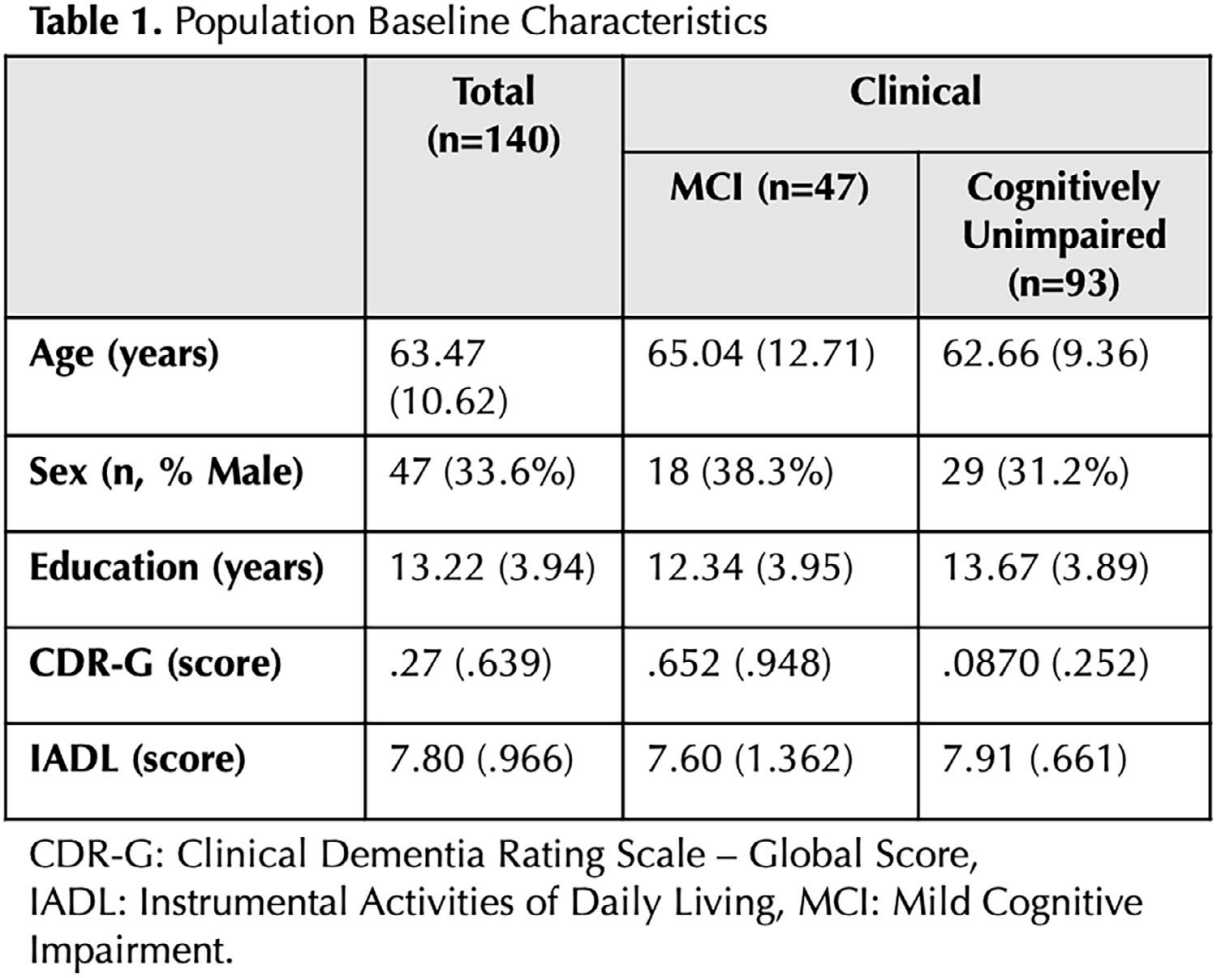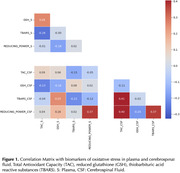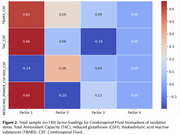# Comparison of cerebrospinal fluid and plasma oxidative stress biomarkers

**DOI:** 10.1002/alz.085482

**Published:** 2025-01-03

**Authors:** Adrian Noriega de la Colina, Sokratis Charisis, Eva Ntanasi, Eirini Mamalaki, Zoi Skaperda, Demetrios Kouretas, Nikolaos Scarmeas

**Affiliations:** ^1^ Montreal Neurological Institute‐Hospital (The Neuro), Montreal, QC Canada; ^2^ McGill University, Montreal, QC Canada; ^3^ Glenn Biggs Institute for Alzheimer’s & Neurodegenerative Diseases, University of Texas Health Sciences Center at San Antonio, San Antonio, TX USA; ^4^ Aiginition Hospital, National and Kapodistrian University of Athens, Medical School, Athens Greece; ^5^ Harokopio University, Athens Greece; ^6^ University of Thessaly, Volos, Thessaly Greece; ^7^ 1st Department of Neurology, Aiginition Hospital, National and Kapodistrian University of Athens Medical School, Athens Greece; ^8^ Taub Institute for Research in Alzheimer’s Disease and the Aging Brain, The Gertrude H. Sergievsky Center, Columbia University Medical Center, New York, NY USA

## Abstract

**Background:**

Numerous studies have highlighted the role of oxidative stress in Alzheimer’s disease (AD) development. Yet, the alignment of systemic and central oxidative stress biomarkers is unclear across diverse populations in the AD continuum. This study aims to assess protein damage levels in plasma and cerebrospinal fluid (CSF) within the AD continuum.

**Methods:**

One hundred forty participants without clinical dementia (47 with Mild Cognitive Impairment [MCI] and 93 cognitively unimpaired) from a memory clinic cohort underwent examination for central (CSF) and systemic (plasma) markers of oxidative stress. We measured Total Antioxidant Capacity (TAC), reduced glutathione (GSH), thiobarbituric acid reactive substances (TBARS), and reducing power (RP) using standard laboratory techniques and absorbance spectrophotometry (Hitachi U‐1500). Pearson’s correlations were employed to determine the congruence between the same biomarkers of oxidative stress in CSF and plasma. Factor analysis was performed to ascertain how many antioxidant dimensions they represent.

**Results:**

Table 1 outlines participants' baseline characteristics. Pearson’s correlation analysis for the entire sample revealed no congruence between CSF and plasma in TAC (r = +.087) and GSH (r = .‐.161), while CSF and plasma RP (r = .+385*) and TBARS (r = ‐.216*) were correlated (Figure 1). Upon analyzing group‐level data, the cognitively unimpaired group showed congruence between CSF and plasma for GSH (r. = ‐.279*) and TBARS (r = ‐.287*) but not for TAC (r = +.191) and RP (r = +.202). In the MCI group, only RP (r. = +.640*) demonstrated congruence between CSF and plasma. Factor analysis with CSF biomarkers identified two components in the whole sample (Figure 2). Dimension 1 = GSH, and Dimension 2 = TAC, TBARS, and RP. Factor analysis with plasma identified as well two components: Dimension 1 = RP and GSH, Dimension 2 = TBARS and TAC. The factor analysis for the MCI group revealed the presence of a single component for CSF biomarkers, while there were two components for plasma: Dimension 1 = RP and TBARS, Dimension 2 = TAC and GSH.

**Conclusion:**

Oxidative stress biomarker patterns differ across the AD continuum. RP demonstrates the most reliability, while TAC is the least. GSH and TBARS plasma and CSF measurements are negatively associated in the cognitively unimpaired, but not in the MCI population. Understanding oxidative biomarker evolution can enhance our comprehension of their role in AD and age‐related disorders.